# Supplementary material for: Learning curve and surgical outcome of robotic assisted colorectal surgery with ERAS program
Source: Sci Rep. 2022 Nov 29;12:20566. doi: 10.1038/s41598-022-24665-w (PMC9709162; doi:10.1038/s41598-022-24665-w)
Supplement: Supplementary file 1 — Supplementary Information 1. [file 41598_2022_24665_MOESM1_ESM.zip › LSC Raw data-Ñ[▒K/102/O_ñw╝╨░O▒Kñσ.pdf]

正式摘要(電子簽章：000000202548967)

請選擇...reporter

臺中榮民總醫院

Operation Note

[redacted]

- 手術日期：2020/11/05
- 手術醫師：VS 蔣鋒帆，CR 林昌霖，CR 林陳彥
- Anesthesia：GE
- Position：Lithotomy
- Aseptic agents：Betadine Alcohol，Betadine Aqua

Diagnosis

- Pre-operative diagnosis : Malignant tumor of sigmoid colon
- Post-operative diagnosis : s/p laparoscopic anterior resection

Surgical Indication

- For removing the primary tumor

Operation

- 1. Intraoperative colonoscopy : (-)
  2. High ligation (+) : At IMA root. IMV and left colic artery were all resected and ligated via hemo-clips.
  3. The colon was not taken down near the splenic flexure
  4. EndoGIA (60-4.1) X2 + EEA (29mm) were used
  5. Proximal cut end : 10 cm, distal cut end : 3 cm
  6. No drainage tube was inserted
  7. The ADHESION BARRIER FILM was placed below the central umbilicus extended wound.

Finding

- 1. A 5 x 5 cm tumor at distal sigmoid colon, which probably invaded over muscular layer. At least cT3.
  2. There was no obvious visible liver metastasis, no visible peritoneal seeding
  3. No obvious ascites was noted
  4. A suspicious small nodule over the mesocolon, near the tumor area. It was resected for tissue sampling.
  5. A small seroa tear over the small bowel was noted. It was strengthened with several interrupted sutures via 3-O mersilk.
  6. Bilateral doughnuts: intact ; Air leak test: Done and no air leak was found.

Procedure

- 1. General endotracheal anesthesia was induced and then the patient was placed in modified Trendelenburg lithotomy position.
  Preoperative antibiotics were given. A Foley catheter and a nasogastric tube were placed. The abdomen was prepped and draped in standard sterile fashion.
  2. A small vertical incision was made right next to the umbilicus and a 12 mm trocar was inserted into the peritoneal cavity by open method. Peumoperitoneum was established with CO2 inflation. Laparoscopy was inserted and then another 12 mm trocar was inserted over RLQ. Extra three 5 mm trochars were inserted over RUQ, LUQ and LLQ of abdomen. The operative findings were mentioned as above.
  3. The small bowel was inspected and retracted to the right by grasper. The sigmoid colon was then held by grasper and the peritoneum overlying the mesentery was then scored with electrocautery. The inferior mesentery artery was identified, ligated below the left colic artery with endoclips, and transected, so was inferior mesentery vein. Dissection was done above the Gerota's fascia and toward the peritoneal attachments.
  4. The arcade vessels to the sigmoid colon were also divided. Distal descending colon and upper rectum were mobilized. Totally two linear staplers (EndoGIA 60-4.1) was then used to divide the rectum.
  5. Incision was further extended from the central umbilicus wound. The proximal colon was then taken out

- for carefully inspection, and to make sure it has an excellent blood supply. The mesenteric fat was gently cleared from the terminal 2-3 mm of the bowel ends.
  6. A suspicious parocolic small nodule was noted. It was resected for smapling.
  7. The field was isolated with moist pad and the bowel opened and sized. It was determined that a 29 mm circular stapler could be accommodated. A pursestring suture of 2-0 prolene was placed, the anvil was inserted, and then the pursestring suture was tied.
  8. The anal canal was irrigated with distil water and then the circular stapler was introduced through the anus and pierced through the mid-portion of the stapler line.
  9. The proximal bowel with anvil in place was engaged with the circular stapler and the stapler was then fired. Two complete donuts were obtained and the distal one was submitted to pathology for margin confirmation.
  10. The anastomosis was tested for leaks with irrigation solution and air introduced through the rectum and was found to be patent and intact.
  11. The possible bleeder was then checked thoroughly.
  12. A small seroa tear over the small bowel was noted. It was strengthened with several interrupted sutures via 3-O mersilk.
  13. The ADHESION BARRIER FILM was then placed below the central extended wound.
  14. The fascia of midline wound was closed with interrupted sutures via Num. 1 Vicryl. The other trochar wounds were closed with interrupted sutures via 1-O Vicryl.
  15. Skin were closed with interrupted 3-0 Vicryl, applied with SurgiSeal and then covered with Medipore.
  16. The patient tolerated the procedure well and was brought to the POR in stable condition.
- Estimated blood loss: minimum
- 器械數量: 正確
- 紗布數量: 正確

Image

- PAINT
  - abdomen

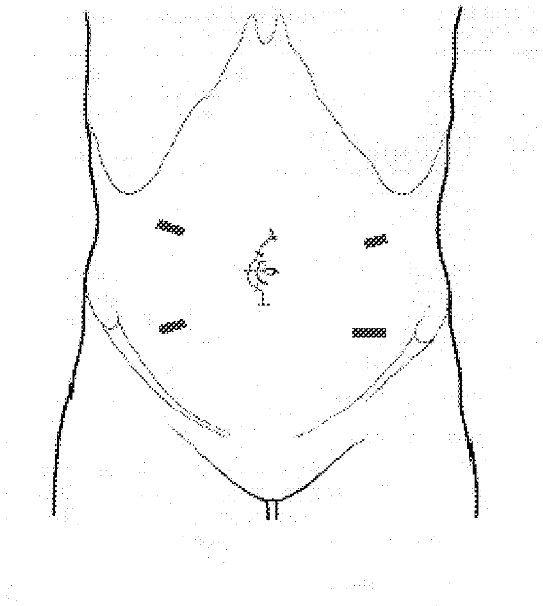

  - colon & rectum

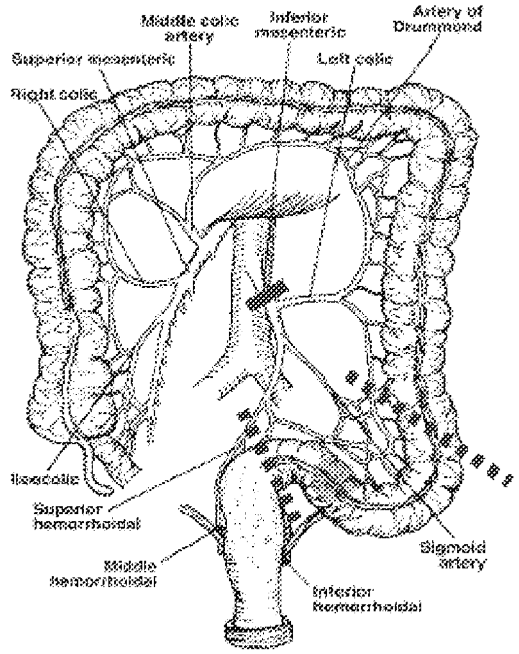

- The resected s-colon

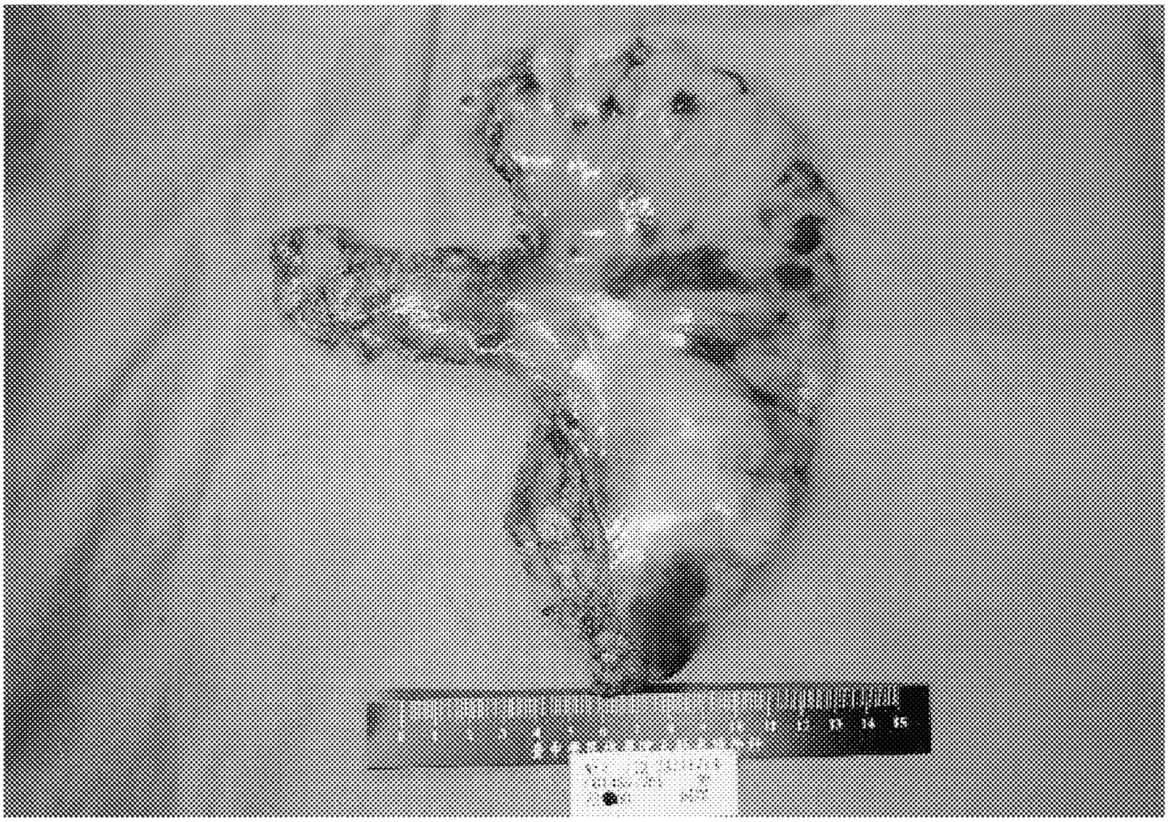

The tumor was presented as below.

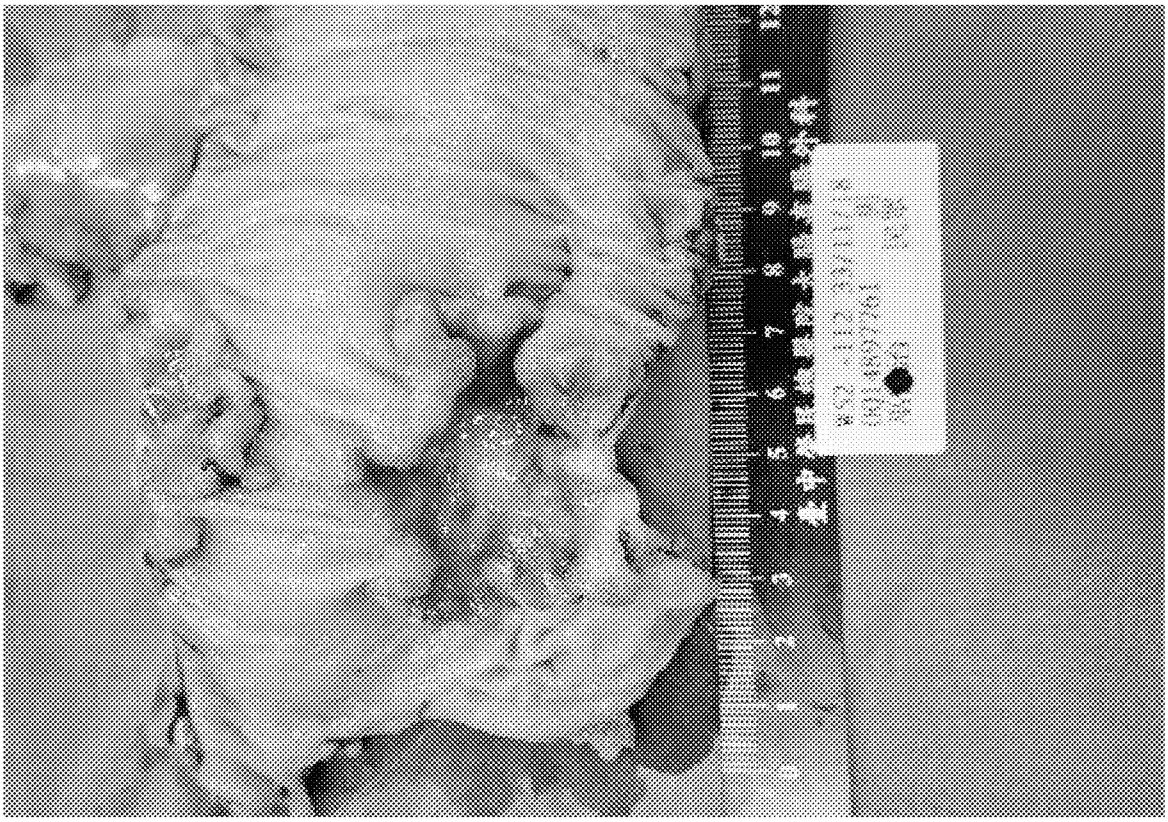

Tumor invasion to serosa. At least cT3.

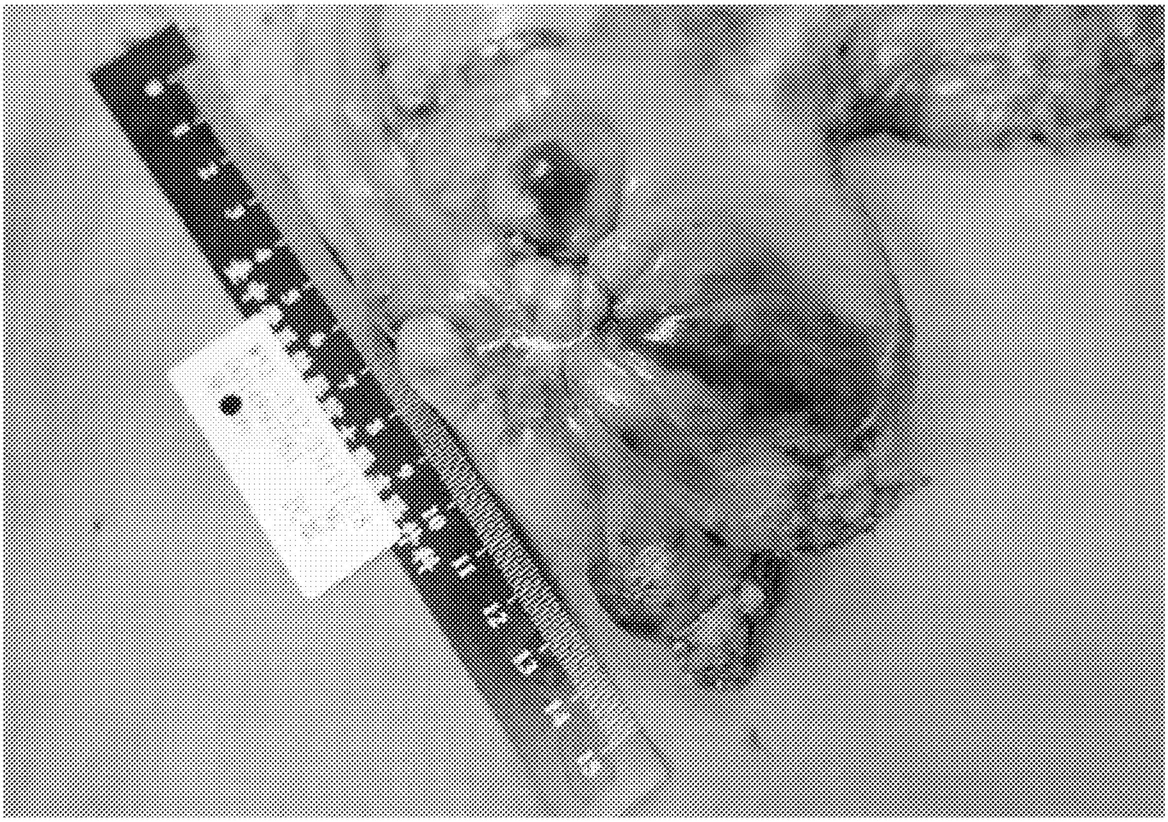

主任 2062F蔣鋒帆 / 醫師 2301H林陳彥

正式摘要(電子簽章：000000202548967)

請選擇...reporter

doctorRank:B
